# Genetics of Human and Canine Dilated Cardiomyopathy

**DOI:** 10.1155/2015/204823

**Published:** 2015-07-22

**Authors:** Siobhan Simpson, Jennifer Edwards, Thomas F. N. Ferguson-Mignan, Malcolm Cobb, Nigel P. Mongan, Catrin S. Rutland

**Affiliations:** ^1^Faculty of Medicine and Health Sciences, School of Veterinary Medicine and Science, The University of Nottingham, Sutton Bonington Campus, Loughborough LE12 5RD, UK; ^2^Department of Pharmacology, Weill Cornell Medical College, 1300 York Avenue, New York, NY 10065, USA

## Abstract

Cardiovascular disease is a leading cause of death in both humans and dogs. Dilated cardiomyopathy (DCM) accounts for a large number of these cases, reported to be the third most common form of cardiac disease in humans and the second most common in dogs. In human studies of DCM there are more than 50 genetic loci associated with the disease. Despite canine DCM having similar disease progression to human DCM studies into the genetic basis of canine DCM lag far behind those of human DCM. In this review the aetiology, epidemiology, and clinical characteristics of canine DCM are examined, along with highlighting possible different subtypes of canine DCM and their potential relevance to human DCM. Finally the current position of genetic research into canine and human DCM, including the genetic loci, is identified and the reasons many studies may have failed to find a genetic association with canine DCM are reviewed.

## 1. Dilated Cardiomyopathy Aetiology and Epidemiology

Cardiovascular disease is the fourth most common cause of death in dogs [1] and one of the most common causes of death in humans [2]. Dilated cardiomyopathy (DCM) is the second most prevalent form of heart disease in dogs, accounting for 10% of cardiac diagnoses [3], and is estimated to be the third most common inherited type of heart disease in humans, reported to affect 35.6 in 100,000 people, although this is thought to be an underestimation [4, 5].

Due to the similar nature of DCM in humans and dogs in terms of disease phenotype and progression, it has been suggested that canine DCM can act as a model for human DCM [6]. Conversely, knowledge obtained from the clinical management of people with DCM may guide improvements in the clinical care and outcomes of companion animals with DCM.

Animal models of DCM are useful in providing insights into the molecular and cellular progression of the disease and thus lead to potential new treatments [7]. While there are many animal models where DCM is induced, for example [8–11], naturally occurring cases of canine DCM are also valuable, in particular with regard to natural disease progression, especially where the underlying cause can be shown to be similar in dogs and people (e.g., similar genetic function) [12]. In addition to providing a potential natural model for human DCM, canine cardiovascular health is an important issue in its own right. Understanding the disorder will impact veterinary care, treatment, and prognosis and may also influence pedigree breeding, health, and welfare. Here we review the clinically distinct types of canine DCM and relate these to clinical heterogeneity seen in human DCM. Furthermore we provide a review of the known genetic contributions to DCM and discuss how these factors may inform future clinical management and breeding strategies in the dog.

## 2. Clinical Characteristics of DCM

DCM is characterised by cardiac ventricular chamber enlargement and systolic dysfunction which often leads to congestive heart failure and death [13]. The aetiology of DCM is complex in that genetic factors, myocardial ischemia, hypertension, toxins, infections, and metabolic defects have been implicated in human disease [14]. Both human and canine DCM have a number of phases of progression starting with a long asymptomatic period before clinical signs appear [6, 15]. During this asymptomatic period, no functional changes in cardiac tissue have yet been reported, but it is possible that the underlying causes (e.g., genetic factors, toxins, and infections) are already initiating the disease [6]. During the next stage, there are again no reported outward clinical signs and the individual usually appears to be healthy, but cardiovascular electrical and morphological changes can be observed [6, 15–17]. Cardiovascular electrical changes may be detected using Holter monitoring for 24 hours, with individuals that go on to develop canine DCM often displaying ventricular arrhythmias [18]. Echocardiography can identify individuals that have an enlarged left ventricle which ultimately leads to symptomatic canine DCM [18]. Due to the apparently asymptomatic nature of this stage it is often termed the occult or preclinical stage and can last for several years in dogs [6, 17, 18]. In the final stage of DCM patients present with clinical signs of heart failure, commonly including cough, depression, dyspnoea, weight loss, and syncope, the individual requires treatment for heart failure, but prognosis is often poor [6, 19]. In humans, mortality 10 years following diagnosis is roughly 40%, although there is a wide variation with some individuals remaining asymptomatic, conversely many individuals suffer from sudden death [20]. Dogs also have significantly shortened lifespan following diagnosis, mean survival time following diagnosis, usually at the point of developing overt clinical symptoms, being 34 weeks, although, similar to humans, large variations are observed, with some surviving for several months while others only live for a few weeks [21–23].

Treatment of DCM in humans is aimed at minimising the effect of heart failure on the patient and delaying disease progression [24]. Standard medical treatment for human DCM consists of ACE inhibitors and *β*-blockade, often with a diuretic agent and, in the latter stages of disease progression, inotropic agents are frequently prescribed [24, 25]. Heart transplants are often the last resort in treating human heart disease; however the proportion of heart transplants in humans due to nonischemic cardiomyopathy, of which DCM is the second most common form, has increased to become the leading cause of heart transplant in recent years: 51% of transplant cases had nonischemic cardiomyopathy [26]. Canine DCM is treated in a similar manner to human DCM, in that treatment is aimed at minimising the effect of heart failure [27]. This treatment usually consists of diuretics, ACE inhibitors, positive inotropes, and other vasodilators [27, 28]. There is evidence that treatment when preclinical symptoms appear can increase lifespan, but this requires screening of individuals for preclinical DCM [29]. Heart transplants and other cardiac assist devices are not generally available to canine DCM patients.

### 2.1. Evidence for Different Types of Canine DCM

Although dogs within all breeds have the potential to develop DCM, there are some breeds that are particularly afflicted by DCM [30]. These include Newfoundlands, St. Bernards, Doberman Pinschers, Great Danes, Irish Wolfhounds, Boxers, and English Cocker Spaniels [3]. While these breeds, as well as other less frequently affected breeds, can be diagnosed as having DCM, there is evidence that different breeds may present with distinct types of DCM. This evidence consists of differential survival times from diagnosis, histopathology, inheritance patterns, and age of onset [21, 31–35].

Within canine DCM, two distinct types of histopathological variations have been described: “attenuated wavy fibre type” and “fatty infiltration type” [32]. While this evidence may be subjective, it adds to the evidence suggesting that there are different types of canine DCM. The fatty infiltration type is less subjective and has only been reported in Doberman Pinschers, Estrela Mountain Dogs, Great Danes, and Boxers [32, 36–38]; while the wavy fibre type is more ubiquitous, it does not seem to be restricted to specific breeds and can also occur in breeds which display the fatty infiltration type [32, 37]. As the wavy fibre type is found across breeds and in many individuals, it could be the tissue's response to the other processes of DCM. In particular atrophy, or attenuation, of muscle fibres is a frequent result of processes that prevent normal contractile ability: contractile ability is consistently compromised in DCM [19]. The prevalence and clinical significance of these histopathological variants remain to be established, although the phenotype can only be established post mortem and thus is unlikely to be useful in a clinical setting.

Human DCM is generally inherited in an autosomal dominant fashion [39], but autosomal recessive, X-linked recessive, and mitochondrial modes of inheritance have all been reported [40]. In common with human inheritance patterns there are several types of inheritance suggested in canine DCM. These include autosomal recessive [34], X-linked [33], and the common autosomal dominant inheritance [35, 38]; although often with reduced penetrance, not all dogs with the DCM genotype will develop the disease [41, 42]. X-linked and autosomal inheritance patterns show that the genetic basis of the disease is different. Recessive and dominant inheritance patterns also suggest the presence of different mutations leading to DCM and reduced penetrance indicates that there are likely to be additional factors involved in the formation of the disease phenotype. These additional factors may involve additional genes, epigenetic effects, and environmental effects including, but not limited to, diet, exercise, stress and toxins, or a combination of any number of these.

There is a wide variation in the long term prognosis of canine DCM. Some dogs, with appropriate disease management, can have a good quality of life for many years following a DCM diagnosis, whereas others die within weeks despite medical intervention [21–23]. Within this variation there are prognosis trends within breeds. Doberman Pinschers are a breed with particularly poor prognosis, and mean time to death (from diagnosis) is in the range of 7.4 to 9.7 weeks [21, 31], while the mean time for other breeds is reported to be about four times that at 34 weeks [21]. Great Danes also suffer from a poor prognosis with Martin et al. [23] finding that they have the lowest median survival time of breeds included in their analysis, while Doberman Pinschers had the lowest upper quartile range.

Age of onset can also affect prognosis. There is a juvenile form of DCM in Portuguese water dogs, where age of onset is measured in weeks from birth [43, 44], while in most other cases age of onset is measured in years [45]. It would seem from this that DCM in Portuguese water dogs is a distinct condition. Even within adult canine DCM there is variation between breeds as to when individuals present with outward clinical signs. For example, Great Dane mean age of onset is 4.8 (SD ± 2.3) years [33], which is comparable to Irish Wolfhound mean age of onset of 4.40 (SD ± 2.03) years [42]; however, Doberman Pinscher's mean age of onset is in 7.3 years in males and 8.6 years in females [31]. This variation in mean age of onset could further suggest that there are different types of canine DCM.

There also appears to be different types of human DCM, with different inheritance patterns and age of onset reported [46]. If canine DCM can be appropriately matched to human DCM in terms of age of onset, inheritance pattern, survival time, and histopathology, they could provide appropriate models for each other. In particular some cases of childhood DCM have been shown to have an autosomal recessive pattern of inheritance [47], and in this instance the juvenile DCM observed in Portuguese water dogs [44] could be an appropriate model. There are currently several types of DCM identified in humans [39], but additional studies of canine DCM phenotypes are required to allow appropriate matching of canine and human DCM categories. Once identified, knowledge about canine DCM types could benefit current and future potential treatments and support for both human and canine DCM patients, in addition to elucidating other clinically important factors in canine DCM, such as longevity and prognosis.

### 2.2. Genomic Research of DCM in Humans

While there are many implicated causes or risk factors related to developing DCM and disease progression, genetics is a common one, with the disease often affecting several individuals within a family. To date mutations in over 50 genes have been associated with DCM in humans; however mutations in the most prevalent DCM related genes only account for approximately 50% of patients with DCM [39]. Genetic testing of individuals related to DCM patients can allow those that are at high risk of developing DCM to be more closely monitored [48]. This genetic testing is carried out on a panel of about 50 loci and more than one locus can be implicated in the disease [14] suggesting a dose effect, whereby the more DCM alleles an individual carries, the more severe the phenotype [39]. Gene penetrance has also been reported to affect disease expression and severity, and likewise the type of mutation and the specific gene which is affected often lead to differing features, age of onset or severity, and prognosis [49, 50].

Human DCM-associated genes identified to date are involved in a range of functions but can usually be placed into one of six functional groups: sarcomeric protein genes, cytoskeletal protein genes, nuclear envelope protein, desmosomal protein genes, calcium/sodium-handling genes, and transcription factor genes [39]. Cardiac muscle consists of striated muscle, and the sarcomere is the smallest unit of contractile muscle within this and thus alterations to this could lead to heart disease [51]. The cytoskeleton forms the majority of the cytoplasm, enabling cells to maintain their shape and facilitating communication within the cell [52, 53]. The nuclear envelope provides a barrier between nucleic acid synthesis and the rest of the cell but must remain permeable to allow the cell to function [54], a large number of proteins within the nuclear envelope have been implicated in chromatin organization and gene regulation [55]. The desmosome provides mechanical strength to tissues and potentially has cell signalling capacity, both of which are essential for cardiac function [56]. Na^+^/Ca^2+^ are important in the contraction of muscle [57] and as such calcium/sodium-handling genes are important in maintaining the correct concentration of Na^+^/Ca^2+^ for contraction of the heart. Transcription factors regulate the rate at which transcription of DNA to mRNA occurs; this rate is important in controlling the expression of genes and therefore the amount of a protein produced [58]. The breakdown of any of these functions has the capacity to lead to disease, including DCM.  Table 1 shows the genes with mutations associated with DCM in humans, including the group into which the gene falls (where appropriate).

### 2.3. Genetics of Canine DCM

Canine DCM has often been used as a model for human DCM, but it is also a major clinical challenge in companion animals [3, 18, 22, 59]. It is has been established that, in common with human DCM, canine DCM frequently has a familial basis [33–35, 42]. Despite this, current understanding of the genetics of canine DCM is limited, in particular compared to the depth of genetic information available for human DCM. Indeed it is only recently that any loci have been associated with canine DCM [6, 60–62]. Genes associated with canine DCM are* DMD* in German short-haired pointers [63],* PDK4* in Doberman Pinschers [60], and* STRN* in Boxers [62], in addition to a locus on chromosome 5 in Doberman Pinschers [6]. Additional polymorphisms on chromosomes 1, 10, 15, 17, 21, and 37 have also been implicated in Irish Wolfhounds [61]. There are two methods that have been employed in attempts to identify genes associated with canine DCM, candidate gene studies, and genome wide association studies (GWAS).

## 3. Canine Candidate Gene Studies

Candidate gene studies for canine DCM primarily involve examining genes with variants associated with human DCM or associated conditions, for example [64–69]. The majority of canine DCM genetic studies have been of this type; however, only one mutation associated with canine DCM has been identified in this manner, which is that of a deletion in the* Striatin* gene in Boxers, a gene previously associated with Boxer arrhythmogenic right ventricular cardiomyopathy using GWAS [62]. All other candidate gene studies have failed to find an association with canine DCM in the cohort examined (see Table 2), and unfortunately the small sample sizes frequently utilised could have limited the power to detect an association. In addition to small sample sizes in a number of studies, control (non-DCM cases) dogs have been limited or have not been appropriate (see Table 2 for exact numbers). Suitable controls should be breed matched and over a certain age to ensure that they are unlikely to develop DCM. Table 2 shows the genes examined for mutations associated with canine DCM in a variety of breeds, sample sizes, and control dogs, in the published literature to date.

## 4. Genome Wide Association Studies (GWAS)

Genome wide association studies are a method of screening the genomes of many individuals for variants or regions that are associated with a trait [70]. Some variants will fall within genes and some outside of genes. When variants associated with a trait are found outside of genes it can be more difficult to establish their mode of action.

There have been three GWAS looking for an association with canine DCM. One of these led to the identification of a deletion in a splice site of* PDK4* associated with DCM in Doberman Pinschers [60]. A separate GWAS in Doberman Pinschers revealed a single SNP associated with DCM in a different location to the* PDK4* gene [6]. The only other GWAS undertaken with regard to canine DCM is that by Philipp et al. [61] which found one significantly associated SNP and five suggestively associated SNPs in Irish Wolfhounds. Of all the loci identified as associated with canine DCM only two are on the same chromosome, one of the Irish Wolfhound SNPs and the* Striatin* genes are both on chromosome 17, but even these are far apart. This indicates that there may be many loci involved in the development of canine DCM.

## 5. The Effects of Multiple Loci on DCM

Thus far in both canine and human genetic DCM studies loci have only been considered for an association with disease individually. There have been indications that multiple loci may influence the development of DCM [6]. In human DCM where a pannel of more than 50 loci are tested concurrently, often several loci are implicated. Simpson et al. [71] have shown theoretically that multiple loci affect the development of DCM in Doberman Pinschers. While this still requires valiadation, it is possible that similar effects occur in other breeds and species.

## 6. Power to Detect an Association with Canine DCM

**Figure 1:**
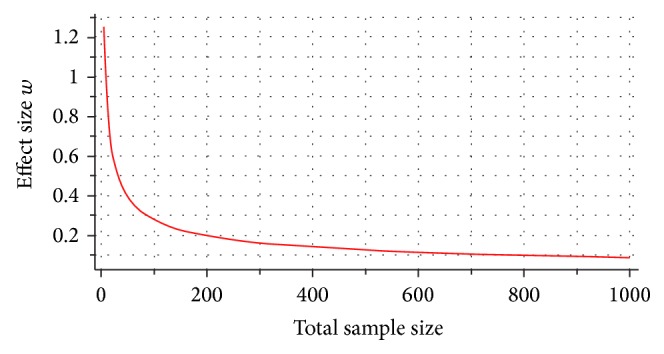
*χ* ^2^ goodness of fit tests: contingency tables Df = 1, *α* err prob = 0.05, and power (1 − *β* err prob) = 0.8.

The majority of studies undertaken with the aim of identifying causal genetic variants of canine DCM have only utilised small samples (5–40 individuals) which is unlikely to be large enough to detect an effect. To establish appropriate study sizes and indicate the effect size that can be detected in published studies G^*∗*^Power 3.1.7 Chi-squared goodness of fit tests were used (using the methods from [72]). This takes known input parameters, including sample size, and calculates estimated effect sizes based on assumed power and can be used to indicate minimum sample size for prescribed power, alpha error rate, and effect size. This was done to indicate minimum sample sizes needed to detect various effect sizes (Figure 1).

Published studies that have identified genetic variants associated with DCM have used sample sizes of 180 [6], 132 [60], and 49 [62]. Assuming these studies had enough power to identify a positive effect (0.8), the effect sizes of these variants in these studies are 0.2088, 0.2438, and 0.4002, respectively, calculated using the sensitivity power analysis in G^*∗*^Power 3.1.7 [72]. These effect sizes, while not large, are larger than the standard effect size for small effect of 0.1. None of these variants explain all incidences of DCM, suggesting that other factors, which may be additional genetic variants of smaller effect, are involved. The sample size required to obtain a positive result from variants with small effect size (0.1) is 785, a number possibly not obtainable for all breeds but could be aimed for in future studies. It is likely that earlier studies concentrated on simple Mendelian recessive, dominant traits and even a multiplicative risk models where Karlsson & Lindblad-Toh [73] had suggested that affected and control groups of 20, 50, and 100, respectively, may suffice. Despite these suggestions, the authors indicated that higher group sizes (around 500 samples) would likely provide sufficient power to map an allele conferring a two-fold risk.

### 6.1. Discussion of Selected Breeds

While there are many breeds affected by canine DCM only a few have had genetic loci identified as associated with the disease. Here we discuss breeds with adolescent and adult onset DCM associated loci. The juvenile DCM that Portuguese water dogs develop is not discussed because it is already considered to be a distinct condition [34].

### 6.2. Boxers: Striatin (*STRN*)

The Boxer breed of dog was developed in the late 1800's primarily from the now extinct hunting dog the Bullenbeisser [74]. As with the development of most modern breeds there is documented evidence of inbreeding to produce the desired characteristics. In the case of the boxer this included a mating of a son to his mother, and following the creation of a breed standard in 1902 it is likely that usually Boxers will have exclusively been mated to other Boxers [74]. This limited genetic diversity is likely to have led to Boxers being prone to developing a number of diseases including heart disease, of which they frequently develop both arrhythmogenic right ventricular cardiomyopathy (ARVC) and DCM [62]. Since boxer cardiomyopathy was described by Harpster [75] there have been several subtypes described, of the two displaying overt clinical symptoms these most closely align to human ARVC and DCM [62]. Recently Meurs et al. [62] tested a deletion in the striatin (*STRN*) gene for an association with DCM in boxers. This deletion has previously been associated with ARVC and it was hypothesised that ARVC and DCM are variants of the same disease in Boxers and the homozygous genotype leads to DCM rather than ARVC [62]. They found a significant association with the deletion in its homozygous form and DCM, but there were three cases of DCM where there was no deletion in the gene, thus indicating that there is at least one more cause of DCM in the breed to be established [62].

### 6.3. Doberman Pinschers:* PDK4* and Chromosome 5 SNP

The Doberman Pinscher breed was developed at the end of the 1800's in Germany [76] when a number of individuals from established breeds were used to improve various characteristics. According to Gruenig [76] these include the Manchester terrier, Greyhound, Rottweiler, Gordon Setter, Old English Sheepdog, Beauceron, Pinscher (probably German Pinscher), Weimaraner, and other less specific breeds such as Mastiff (possibly Great Dane), Hound, and Sporting dogs. The development of the breed happened rapidly, over a period of about 30 years, and since then Doberman Pinschers have only been bred to Doberman Pinschers [76], leading to a closed gene pool. Although a number of breeds contributed to the Doberman Pinscher it is likely that relatively few individuals of each breed were used likely leading to low genetic diversity. In addition to relatively few founders there is evidence of some individuals contributing a greater number of offspring to the breeding population than others [76].

Doberman Pinschers can develop a particularly severe type of DCM with rapid disease progression following the diagnosis of DCM with mean survival time of less than 10 weeks [21, 31]. Poor survival time following diagnosis combined with the high prevalence of the disease with estimates ranging from 45% to 63% means DCM in this breed is a particular problem for clinicians [59]. Doberman Pinschers display the fatty infiltration type of histopathology [32]. Despite these poor statistics, age of onset of clinical signs is often later than in other commonly affected breeds (7.3 years in males and 8.6 years in females, compared to 4.8 (SD ± 2.3) years in Great Danes), giving individuals a good quality of life up until overt DCM clinical signs [31, 77]. Across age groups there is no difference in clinical signs associated with DCM between the sexes including echocardiographic changes, presence and number of ventricular premature contractions, and overt DCM [59]. Unfortunately, however, males are more likely to have overt DCM than females with 73.7% of all observed males becoming clinically overt while only 26.3% of females observed became clinically overt [59].

DCM in Doberman Pinschers appears to be inherited in an autosomal dominant fashion with equal numbers of males and females affected, male-male transmission, and the mating of two affected individuals producing unaffected offspring [35]. There have been two loci identified as associated with DCM in the breed, a deletion of a splice site in pyruvate dehydrogenase kinase, isozyme 4 (*PDK4*), and a SNP on chromosome 5 [6, 60]. Unfortunately neither of these loci explains all incidences of DCM, and the PDK4 deletion is not significantly associated with DCM in a separate Doberman Pinscher population [78]. There are still additional causes of DCM to be identified in Doberman Pinschers and the function of the SNP on chromosome 5 needs to be established.

### 6.4. German Short-Haired Pointers: Dystrophin (*DMD*)

The only gene associated with canine DCM in German short-haired pointers is Dystrophin (*DMD*) [63]. German short-haired pointers are not considered a breed particularly afflicted by heart disease and the deletion was only identified in two male litter mates [3, 63]. This could be an isolated case which is unlikely to have implications in other breeds, particularly as the affected individuals also had skeletal myopathies, whereas in most cases of canine DCM there are not any other myopathies present [63].

### 6.5. Irish Wolfhounds

Although Irish Wolfhounds have a long history, this includes a period when they were close to extinction. As part of conserving the breed, Great Danes, Scottish deerhounds, Borzoi, and Mastiffs were crossed with the few remaining Irish Wolfhounds [61, 79]. While this will have introduced some degree of genetic diversity to the breed, by necessity a large amount of inbreeding will have been required to retain the Irish Wolfhound phenotype and so, like most modern breeds, genetic diversity is low [80].

Irish Wolfhounds do not usually develop a particularly severe form of DCM and with appropriate management can live with the disease for many months or years [22]. Unfortunately, however, the prevalence of heart disease, including DCM, within the breed is very high, with 41% of individuals presenting with cardiac abnormalities, of which 58% have DCM [22]. This high prevalence combined with early onset of clinical signs at around 4 years old [42] means that DCM in Irish Wolfhounds is of concern and so identifying genetic causes of the disease could have a large impact on the health of breed.

The mode of inheritance of DCM in Irish Wolfhounds has been shown to be autosomal dominant major gene effect, but with reduced penetrance indicating that multiple factors influence disease progression [42]. Of the six SNPs associated with DCM in Irish Wolfhounds to date, only three lie within known genes [61]. Further work is therefore required to establish the functional significance of the alleles and to confirm the associations with DCM.

## 7. Conclusions:* Impact of Genetics on Canine DCM*

In the short term, the identification of the genetic contributors to DCM will enable targeted heart monitoring prior to the onset of clinical signs and clinical management of those dogs with increased risk of developing DCM. In the longer term, knowledge of the genetic factors which predispose to DCM will allow for selective breeding strategies to be considered and may identify novel therapeutic and diagnostic approaches. Individuals likely to develop DCM, identified through robust genetics, could be removed from breeding programmes with the ultimate goal of reducing the number of affected animals within the population and promoting the long term welfare of the breed. Understanding the genetic causes may also aid the stratification of distinct clinical subtypes of DCM. This knowledge may also permit the development of novel DCM management programmes, help to guide prognosis, and assist with future drug and intervention research. Furthermore, investigations into causative genes in canine DCM may prove beneficial for other species, including humans. Novel mutations in canine breeds may serve as candidate genes in affected humans. For these reasons a more detailed understanding of the genetic basis of DCM in diverse dog breeds is now required.

## Figures and Tables

**Table 1:** Genes with mutations associated with DCM in humans.

Gene	Location/role	Reference
ABCC9	Calcium/sodium-handling	[81]
ACTC1	Sarcomere & cytoskeleton	[82]
ACTN2	Sarcomere & cytoskeleton	[83]
ANKRD1	Sarcomere & transcription factor	[84]
BAG3	Sarcomere	[85–87]
CAV3	Other	[88]
CHRM2	Other	[89]
CRYAB	Cytoskeleton	[90]
CSRP3	Sarcomere & cytoskeleton	[83]
CTF1	Other	[91]
DES	Cytoskeleton	[92, 93]
DMD	Cytoskeleton	[94, 95]
DNAJC19	Other	[96]
DOLK	Other	[97]
DSC2	Desmosome	[98]
DSG2	Desmosome	[99]
DSP	Desmosome	[100]
EYA4	Other	[101]
FHL2	Sarcomere & cytoskeleton	[102]
FKTN	Cytoskeleton	[103]
FKRP	Cytoskeleton	[104]
FOXD4	Transcription factor	[105]
GATAD1	Other	[106]
HCG22	Other	[107]
HLA-DQB1	Other	[108]
HSPB7	Other	[109]
ILK	Cytoskeleton	[110]
LAMA2	Other	[111]
LAMA4	Cytoskeleton	[110]
LAMP2	Other	[112]
LDB3	Sarcomere & cytoskeleton	[113]
LMNA	Nuclear envelope	[114]
MURC	Other	[115]
MYBPC3	Sarcomere	[116, 117]
MYH6	Sarcomere	[117, 118]
MYH7	Sarcomere	[116]
MYPN	Cytoskeleton	[119]
NEBL	Sarcomere	[120]
NEXN	Sarcomere	[121]
NOS3	Other	[122]
PKP2	Desmosome	[98]
PLN	Calcium/sodium-handling	[123]
PRDM16	Transcription factor	[124]
PSEN1	Other	[125]
PSEN2	Other	[125]
RBM20	Other	[126]
RYR2	Calcium/sodium-handling	[127]
SCN5A	Calcium/sodium-handling	[128]
SDHA	Other	[129]
SGCD	Cytoskeleton	[130]
SYNE1	Nuclear envelope	[131]
TAZ	Other	[132]
TBX20	Transcription factor	[133]
TCAP	Sarcomere & cytoskeleton	[134]
TMPO	Nuclear envelope	[135]
TNNC1	Sarcomere	[117]
TNNI3	Sarcomere	[136]
TNNT2	Sarcomere	[137]
TPM1	Sarcomere	[117]
TXNRD2	Other	[138]
TTN	Sarcomere & cytoskeleton	[139]
VCL	Sarcomere & cytoskeleton	[140]
ZBTB17	Other	[141]

**Table 2:** All genes investigated in relation to canine DCM.

Gene	Associated with DCM in humans	Associated with canine DCM	DCM dogs		Control dogs	Human reference	Canine study reference
			Number	Breed			
ACTC1	Y	N	16	Doberman Pinscher	12 mixed breeds	[39, 142]	[77]
			64	Irish Wolfhound	25 Irish Wolfhounds		[143]
			38	Newfoundland	36 Newfoundlands		[69]
			5	Doberman Pinscher	5 unspecified dogs without overt heart disease		[144]

ACTN2	Y	N	5	Doberman Pinscher	5 unspecified dogs without overt heart disease	[39, 142]	[144]

CAV1	N	N	38	Newfoundland	36 Newfoundlands	na	[69]

CSRP3	Y	N	64	Irish Wolfhound	25 Irish Wolfhounds	[39, 142]	[143]
			38	Newfoundland	36 Newfoundlands		[69]
			5	Doberman Pinscher	2 Labradors		[67]
			5	Doberman Pinscher	5 unspecified dogs without overt heart disease		[144]

DES	Y	N	25	Doberman Pinscher	10 Doberman Pinschers	[39, 142]	[35]
			64	Irish Wolfhound	25 Irish Wolfhounds		[143]
			18	Doberman Pinscher	10 Doberman Pinschers		[68]
			38	Newfoundland	36 Newfoundlands		[69]

DMD	Y	Y	2	German short-haired pointers	2 German short-haired pointers with reduced dystrophin	[39, 142]	[63]

LDB3	Y	N	38	Newfoundland	36 Newfoundlands	[39, 142]	[69]

LMNA	Y	N	38	Newfoundland	36 Newfoundlands	[39, 142]	[69]
			5	Doberman Pinscher	2 Labradors		[67]

MYBPC3	Y	N	5	Doberman Pinscher	5 unspecified dogs without overt heart disease	[39, 142]	[144]

MYH7	Y	N	38	Newfoundland	36 Newfoundlands	[39, 142]	[69]
			5	Doberman Pinscher	2 Labradors		[67]
			5	Doberman Pinscher	5 unspecified dogs without overt heart disease		[144]

PDK4	N	Y	66	Doberman Pinscher	66 Doberman Pinschers + 100 others from 11 breeds	na	[60]

PLN	Y	N	25	Doberman Pinscher	10 Doberman Pinschers	[39, 142]	[35]
			2 Doberman Pinscher, 2 Newfoundland, 2 great dane		computer database only		[66]
			64	Irish Wolfhound	25 Irish Wolfhounds		[143]
			38	Newfoundland	36 Newfoundlands		[69]

SGCD	Y	N	25	Doberman Pinscher	10 Doberman Pinschers	[39, 142]	[35]
			64	Irish Wolfhound	25 Irish Wolfhounds		[143]
			38	Newfoundland	36 Newfoundlands		[69]
			25	Doberman Pinscher	13 Doberman Pinschers		[145]

STRN	N	Y	33	Boxer	16 Boxers	na	[62]

TAZ	Y	N	64	Irish Wolfhound	25 Irish Wolfhounds	[39]	[143]

TCAP	Y	N	8	Irish Wolfhound	5 Irish Wolfhounds	[39, 142]	[146]
			38	Newfoundland	36 Newfoundlands		[69]
			5	Doberman Pinscher	5 unspecified dogs without overt heart disease		[144]

TMOD	N	N	64	Irish Wolfhound	25 Irish Wolfhounds	na	[143]

TNNC1	Y	N	5	Doberman Pinscher	2 Labradors	[39, 142]	[67]

TNNI3	Y	N	38	Newfoundland	36 Newfoundlands	[39, 142]	[69]

TNNT2	Y	N	5	Doberman Pinscher	2 Labradors	[39, 142]	[67]
			5	Doberman Pinscher	5 dogs without overt heart disease		[144]
			38	Newfoundland	36 Newfoundlands		[69]

TPM1	Y	N	38	Newfoundland	36 Newfoundlands	[39]	[69]
			5	Doberman Pinscher	5 dogs without overt heart disease		[144]

TTN	Y	N	38	Newfoundland	36 Newfoundlands	[39, 142]	[69]
			2	Doberman Pinscher	5 mixed breeds		[147]

VCL	Y	N	5	Doberman Pinscher	5 dogs without overt heart disease	[39, 142]	[144]
			38	Newfoundland	36 Newfoundlands		[69]

Y = yes, N = no.

## References

[1] Fleming J. M., Creevy K. E., Promislow D. E. L. (2011). Mortality in north american dogs from 1984 to 2004: an investigation into age-, size-, and breed-related causes of death. *Journal of Veterinary Internal Medicine*.

[2] Mathers C. D., Loncar D. (2006). Projections of global mortality and burden of disease from 2002 to 2030. *PLoS Medicine*.

[3] Egenvall A., Bonnett B. N., Häggström J. (2006). Heart disease as a cause of death in insured Swedish dogs younger than 10 years of age. *Journal of Veterinary Internal Medicine*.

[4] Raju H., Alberg C., Sagoo G. S., Burton H., Behr E. R. (2011). Inherited cardiomyopathies. *British Medical Journal*.

[5] Hershberger R. E., Morales A., Siegfried J. D. (2010). Clinical and genetic issues in dilated cardiomyopathy: a review for genetics professionals. *Genetics in Medicine*.

[6] Mausberg T.-B., Wess G., Simak J. (2011). A locus on chromosome 5 is associated with dilated cardiomyopathy in doberman pinschers. *PLoS ONE*.

[7] Dixon J. A., Spinale F. G. (2009). Large animal models of heart failure: a critical link in the translation of basic science to clinical practice. *Circulation: Heart Failure*.

[8] Akar F. G., Wu R. C., Juang G. J. (2005). Molecular mechanisms underlying K^+^ current downregulation in canine tachycardia-induced heart failure. *The American Journal of Physiology—Heart and Circulatory Physiology*.

[9] Argenziano M., Dickstein M. L. (2000). Cardiovascular effects of inhaled nitric oxide in a canine model of cardiomyopathy. *The Annals of Thoracic Surgery*.

[10] Toyoda Y., Okada M., Kashem M. A. (1998). A canine model of dilated cardiomyopathy induced by repetitive intracoronary doxorubicin administration. *Journal of Thoracic and Cardiovascular Surgery*.

[11] Huntington K., Picard P., Moe G., Stewart D. J., Albernaz A., Monge J. C. (1998). Increased cardiac and pulmonary endothelin-1 mRNA expression in canine pacing-induced heart failure. *Journal of Cardiovascular Pharmacology*.

[12] Hongo M., Ryoke T., Ross J. (1997). Animal models of heart failure: recent developments and perspectives. *Trends in Cardiovascular Medicine*.

[13] Guttmann O. P., Mohiddin S. A., Elliott P. M. (2014). Almanac 2014: cardiomyopathies. *Heart*.

[14] McNally E. M., Golbus J. R., Puckelwartz M. J. (2013). Genetic mutations and mechanisms in dilated cardiomyopathy. *The Journal of Clinical Investigation*.

[15] Mahon N. G., Murphy R. T., MacRae C. A., Caforio A. L., Elliott P. M., McKenna W. J. (2005). Echocardiographic evaluation in asymptomatic relatives of patients with dilated cardiomyopathy reveals preclinical disease. *Annals of Internal Medicine*.

[16] Baig M. K., Goldman J. H., Caforio A. L. P., Coonar A. S., Keeling P. J., McKenna W. J. (1998). Familial dilated cardiomyopathy: cardiac abnormalities are common in asymptomatic relative and may represent early disease. *Journal of the American College of Cardiology*.

[17] Brownlie S. E., Cobb M. A. (1999). Observations on the development of congestive heart failure in Irish wolfhounds with dilated cardiomyopathy. *Journal of Small Animal Practice*.

[18] Dukes-McEwan J., Borgarelli M., Tidholm A., Vollmar A. C., Häggström J. (2003). Proposed guidelines for the diagnosis of canine idiopathic dilated cardiomyopathy. *Journal of Veterinary Cardiology*.

[19] Tidholm A., Häggström J., Borgarelli M., Tarducci A. (2001). Canine idiopathic dilated cardiomyopathy. Part I: Aetiology, clinical characteristics, epidemiology and pathology,. *Veterinary Journal*.

[20] Lehrke S., Lossnitzer D., Schöb M. (2011). Use of cardiovascular magnetic resonance for risk stratification in chronic heart failure: prognostic value of late gadolinium enhancement in patients with non-ischaemic dilated cardiomyopathy. *Heart*.

[21] Petric A. D., Stabej P., Zemva A. (2002). Dilated cardiomyopathy in doberman pinschers: survival, causes of death and a pedigree review in a related line. *Journal of Veterinary Cardiology*.

[22] Vollmar A. C. (2000). The prevalence of cardiomyopathy in the Irish wolfhound: a clinical study of 500 dogs. *Journal of the American Animal Hospital Association*.

[23] Martin M. W. S., Johnson M. J. S., Strehlau G., King J. N. (2010). Canine dilated cardiomyopathy: a retrospective study of prognostic findings in 367 clinical cases. *Journal of Small Animal Practice*.

[24] Jefferies J. L., Towbin J. A. (2010). Dilated cardiomyopathy. *The Lancet*.

[25] Merlo M., Pivetta A., Pinamonti B. (2014). Long-term prognostic impact of therapeutic strategies in patients with idiopathic dilated cardiomyopathy: changing mortality over the last 30 years. *European Journal of Heart Failure*.

[26] Taylor D. O., Stehlik J., Edwards L. B. (2009). Registry of the international society for heart and lung transplantation: twenty-sixth official adult heart transplant report-2009. *Journal of Heart and Lung Transplantation*.

[27] McEwan J. D. (2000). Canine dilated cardiomyopathy 2. Pathophysiology and treatment. *In Practice*.

[28] Borgarelli M., Tarducci A., Tidholm A., Häggström J. (2001). Canine idiopathic dilated cardiomyopathy. Part II: pathophysiology and therapy. *Veterinary Journal*.

[29] Summerfield N. J., Boswood A., O'Grady M. R. (2012). Efficacy of pimobendan in the prevention of congestive heart failure or sudden death in Doberman Pinschers with preclinical dilated cardiomyopathy (the PROTECT Study). *Journal of Veterinary Internal Medicine*.

[30] Tidholm A., Jönsson L. (1997). A retrospective study of canine dilated cardiomyopathy (189 cases). *Journal of the American Animal Hospital Association*.

[31] Calvert C. A., Pickus C. W., Jacobs G. J., Brown J. (1997). Signalment, survival, and prognostic factors in Doberman pinschers with end-stage cardiomyopathy. *Journal of Veterinary Internal Medicine*.

[32] Tidholm A., Jönsson L. (2005). Histologic characterization of canine dilated cardiomyopathy. *Veterinary Pathology*.

[33] Meurs K. M., Miller M. W., Wright N. A. (2001). Clinical features of dilated cardiomyopathy in Great Danes and results of a pedigree analysis: 17 cases (1990–2000). *Journal of the American Veterinary Medical Association*.

[34] Sleeper M. M., Henthorn P. S., Vijayasarathy C. (2002). Dilated cardiomyopathy in juvenile Portuguese water dogs. *Journal of Veterinary Internal Medicine*.

[35] Meurs K. M., Fox P. R., Norgard M. (2007). A prospective genetic evaluation of familial dilated cardiomyopathy in the Doberman pinscher. *Journal of Veterinary Internal Medicine*.

[36] Lobo L., Carvalheira J., Canada N., Bussadori C., Gomes J. L., Faustino A. M. R. (2010). Histologic characterization of dilated cardiomyopathy in Estrela mountain dogs. *Veterinary Pathology*.

[37] Aupperle H., Marz I., Baldauf K., Roggon N., Kresken J. G. (2014). Pathology of DCM in Great Danes. *Journal of Veterinary Internal Medicine*.

[38] Stephenson H. M., Fonfara S., López-Alvarez J., Cripps P., Dukes-McEwan J. (2012). Screening for dilated cardiomyopathy in Great Danes in the United Kingdom. *Journal of Veterinary Internal Medicine*.

[39] Posafalvi A., Herkert J. C., Sinke R. J. (2013). Clinical utility gene card for: dilated cardiomyopathy (CMD). *European Journal of Human Genetics*.

[40] Dec G. W., Fuster V. (1994). Idiopathic dilated cardiomyopathy. *The New England Journal of Medicine*.

[41] Dukes-McEwan J., Jackson I. J. (2002). The promises and problems of linkage analysis by using the current canine genome map. *Mammalian Genome*.

[42] Distl O., Vollmar A. C., Broschk C., Hamann H., Fox P. R. (2007). Complex segregation analysis of dilated cardiomyopathy (DCM) in Irish wolfhounds. *Heredity*.

[43] Werner P., Raducha M. G., Prociuk U., Sleeper M. M., van Winkle T. J., Henthorn P. S. (2008). A novel locus for dilated cardiomyopathy maps to canine chromosome 8. *Genomics*.

[44] Dambach D. M., Lannon A., Sleeper M. M., Buchanan J. (1999). Familial dilated cardiomyopathy of young Portuguese water dogs. *Journal of Veterinary Internal Medicine*.

[45] Martin M. W. S., Stafford Johnson M. J., Celona B. (2009). Canine dilated cardiomyopathy: a retrospective study of signalment, presentation and clinical findings in 369 cases. *Journal of Small Animal Practice*.

[46] Mestroni L., Rocco C., Gregori D. (1999). Familial dilated cardiomyopathy: evidence for genetic and phenotypic heterogeneity. *Journal of the American College of Cardiology*.

[47] Towbin J. A., Lowe A. M., Colan S. D. (2006). Incidence, causes, and outcomes of dilated cardiomyopathy in children. *Journal of the American Medical Association*.

[48] Jacoby D., McKenna W. J. (2012). Genetics of inherited cardiomyopathy. *European Heart Journal*.

[49] Mestroni L., Taylor M. R. G. (2013). Genetics and genetic testing of dilated cardiomyopathy: a new perspective. *Discovery Medicine*.

[50] Morales A., Hershberger R. E. (2013). Genetic evaluation of dilated cardiomyopathy. *Current Cardiology Reports*.

[51] Hamdani N., Kooij V., van Dijk S. (2008). Sarcomeric dysfunction in heart failure. *Cardiovascular Research*.

[52] Frixione E. (2000). Recurring views on the structure and function of the cytoskeleton: a 300-year epic. *Cell Motility and the Cytoskeleton*.

[53] Hein S., Kostin S., Heling A., Maeno Y., Schaper J. (2000). The role of the cytoskeleton in heart failure. *Cardiovascular Research*.

[54] Macara I. G. (2001). Transport into and out of the nucleus. *Microbiology and Molecular Biology Reviews*.

[55] Hetzer M. W. (2010). The nuclear envelope. *Cold Spring Harbor Perspectives in Biology*.

[56] Green K. J., Gaudry C. A. (2000). Are desmosomes more than tethers for intermediate filaments?. *Nature Reviews Molecular Cell Biology*.

[57] DiPolo R., Beaugé L. (2006). Sodium/calcium exchanger: influence of metabolic regulation on ion carrier interactions. *Physiological Reviews*.

[58] Latchman D. S. (1997). Transcription factors: an overview. *The International Journal of Biochemistry & Cell Biology*.

[59] Wess G., Schulze A., Butz V. (2010). Prevalence of dilated cardiomyopathy in Doberman Pinschers in various age groups. *Journal of Veterinary Internal Medicine*.

[60] Meurs K. M., Lahmers S., Keene B. W. (2012). A splice site mutation in a gene encoding for PDK4, a mitochondrial protein, is associated with the development of dilated cardiomyopathy in the Doberman pinscher. *Human Genetics*.

[61] Philipp U., Vollmar A., Häggström J., Thomas A., Distl O. (2012). Multiple loci are associated with dilated cardiomyopathy in irish wolfhounds. *PLoS ONE*.

[62] Meurs K. M., Stern J. A., Sisson D. D. (2013). Association of dilated cardiomyopathy with the striatin mutation genotype in boxer dogs. *Journal of Veterinary Internal Medicine*.

[63] Schatzberg S. J., Olby N. J., Breen M. (1999). Molecular analysis of a spontaneous dystrophin ‘knockout’ dog. *Neuromuscular Disorders*.

[64] Philipp U., Broschk C., Vollmar A., Distl O. (2007). Evaluation of tafazzin as candidate for dilated cardiomyopathy in Irish wolfhounds. *Journal of Heredity*.

[65] Wiersma A. C., Leegwater P. A., van Oost B. A., Ollier W. E., Dukes-McEwan J. (2007). Canine candidate genes for dilated cardiomyopathy: annotation of and polymorphic markers for 14 genes. *BMC Veterinary Research*.

[66] Stabej P., Leegwater P. A., Stokhof A. A., Domanjko-Petrič A., van Oost B. A. (2005). Evaluation of the phospholamban gene in purebred large-breed dogs with dilated cardiomyopathy. *American Journal of Veterinary Research*.

[67] Meurs K. M., Hendrix K. P., Norgard M. M. (2008). Molecular evaluation of five cardiac genes in Doberman Pinschers with dilated cardiomyopathy. *American Journal of Veterinary Research*.

[68] Stabej P., Imholz S., Versteeg S. A. (2004). Characterization of the canine desmin (DES) gene and evaluation as a candidate gene for dilated cardiomyopathy in the Dobermann. *Gene*.

[69] Wiersma A. C., Stabej P., Leegwater P. A. J., van Oost B. A., Ollier W. E., Dukes-McEwan J. (2008). Evaluation of 15 candidate genes for dilated cardiomyopathy in the Newfoundland dog. *Journal of Heredity*.

[70] Manolio T. A. (2010). Genomewide association studies and assessment of the risk of disease. *The New England Journal of Medicine*.

[71] Simpson S., Edwards J., Emes R. D., Cobb M. A., Mongan N. P., Rutland C. S. (2015). A predictive model for canine dilated cardiomyopathy—a meta-analysis of Doberman Pinscher data. *PeerJ*.

[72] Faul F., Erdfelder E., Buchner A., Lang A.-G. (2009). Statistical power analyses using G^*^ power 3.1: tests for correlation and regression analyses. *Behavior Research Methods*.

[73] Karlsson E. K., Lindblad-Toh K. (2008). Leader of the pack: gene mapping in dogs and other model organisms. *Nature Reviews Genetics*.

[74] Wagner J. P. (1939). *The Boxer*.

[75] Harpster N. (1983). Boxer cardiomyopathy. *Current Veterinary Therapy VIII*.

[76] Gruenig P. (1939). *The Doberman Pinscher: History and Development of the Breed*.

[77] Meurs K. M., Magnon A. L., Spier A. W., Miller M. W., Lehmkuhl L. B., Towbin J. A. (2001). Evaluation of the cardiac actin gene in Doberman Pinschers with dilated cardiomyopathy. *American Journal of Veterinary Research*.

[78] Owczarek-Lipska M., Mausberg T.-B., Stephenson H., Dukes-McEwan J., Wess G., Leeb T. (2013). A 16-bp deletion in the canine PDK4 gene is not associated with dilated cardiomyopathy in a European cohort of Doberman Pinschers. *Animal Genetics*.

[79] Samaha J. (1991). *The New Complete Irish Wolfhound*.

[80] Jansson M., Laikre L. (2014). Recent breeding history of dog breeds in Sweden: modest rates of inbreeding, extensive loss of genetic diversity and lack of correlation between inbreeding and health. *Journal of Animal Breeding and Genetics*.

[81] Bienengraeber M., Olson T. M., Selivanov V. A. (2004). ABCC9 mutations identified in human dilated cardiomyopathy disrupt catalytic KATP channel gating. *Nature Genetics*.

[82] Olson T. M., Michels V. V., Thibodeau S. N., Tai Y.-S., Keating M. T. (1998). Actin mutations in dilated cardiomyopathy, a heritable form of heart failure. *Science*.

[83] Mohapatra B., Jimenez S., Lin J. H. (2003). Mutations in the muscle LIM protein and *α*-actinin-2 genes in dilated cardiomyopathy and endocardial fibroelastosis. *Molecular Genetics and Metabolism*.

[84] Moulik M., Vatta M., Witt S. H. (2009). ANKRD1, the gene encoding cardiac ankyrin repeat protein, is a novel dilated cardiomyopathy gene. *Journal of the American College of Cardiology*.

[85] Norton N., Li D., Rieder M. J. (2011). Genome-wide studies of copy number variation and exome sequencing identify rare variants in *BAG3* as a cause of dilated cardiomyopathy. *The American Journal of Human Genetics*.

[86] Villard E., Perret C., Gary F. (2011). A genome-wide association study identifies two loci associated with heart failure due to dilated cardiomyopathy. *European Heart Journal*.

[87] Arimura T., Ishikawa T., Nunoda S., Kawai S., Kimura A. (2011). Dilated cardiomyopathy-associated BAG3 mutations impair Z-disc assembly and enhance sensitivity to apoptosis in cardiomyocytes. *Human Mutation*.

[88] Catteruccia M., Sanna T., Santorelli F. M. (2009). Rippling muscle disease and cardiomyopathy associated with a mutation in the CAV3 gene. *Neuromuscular Disorders*.

[89] Zhang L., Hu A., Yuan H. (2008). A missense mutation in the CHRM2 gene is associated with familial dilated cardiomyopathy. *Circulation Research*.

[90] Inagaki N., Hayashi T., Arimura T. (2006). *α*B-crystallin mutation in dilated cardiomyopathy. *Biochemical and Biophysical Research Communications*.

[91] Erdmann J., Hassfeld S., Kallisch H., Fleck E., Regitz-Zagrose V. (2000). Genetic variants in the promoter (g983G>T) and coding region (A92T) of the human cardiotrophin-1 gene (CTF1) in patients with dilated cardiomyopathy. *Human Mutation*.

[92] Schaper J., Froede R., Hein S. (1991). Impairment of the myocardial ultrastructure and changes of the cytoskeleton in dilated cardiomyopathy. *Circulation*.

[93] Li D., Tapscoft T., Gonzalez O. (1999). Desmin mutation responsible for idiopathic dilated cardiomyopathy. *Circulation*.

[94] Muntoni F., Cau M., Ganau A. (1993). Brief report: deletion of the dystrophin muscle-promoter region associated with X-linked dilated cardiomyopathy. *The New England Journal of Medicine*.

[95] Ortiz-Lopez R., Li H., Su J., Goytia V., Towbin J. A. (1997). Evidence for a dystrophin missense mutation as a cause of X-linked dilated cardiomyopathy. *Circulation*.

[96] Davey K. M., Parboosingh J. S., McLeod D. R. (2006). Mutation of DNAJC19, a human homologue of yeast inner mitochondrial membrane co-chaperones, causes DCMA syndrome, a novel autosomal recessive Barth syndrome-like condition. *Journal of Medical Genetics*.

[97] Lefeber D. J., de Brouwer A. P. M., Morava E. (2011). Autosomal recessive dilated cardiomyopathy due to DOLK mutations results from abnormal dystroglycan O-mannosylation. *PLoS Genetics*.

[98] Elliott P., O'Mahony C., Syrris P. (2010). Prevalence of desmosomal protein gene mutations in patients with dilated cardiomyopathy. *Circulation: Cardiovascular Genetics*.

[99] Posch M. G., Posch M. J., Geier C. (2008). A missense variant in desmoglein-2 predisposes to dilated cardiomyopathy. *Molecular Genetics and Metabolism*.

[100] Norgett E. E., Hatsell S. J., Carvajal-Huerta L. (2000). Recessive mutation in desmoplakin disrupts desmoplakin-intermediate filament interactions and causes dilated cardiomyopathy, woolly hair and keratoderma. *Human Molecular Genetics*.

[101] Schönberger J., Wang L., Shin J. T. (2005). Mutation in the transcriptional coactivator EYA4 causes dilated cardiomyopathy and sensorineural hearing loss. *Nature Genetics*.

[102] Arimura T., Hayashi T., Matsumoto Y. (2007). Structural analysis of four and half LIM protein-2 in dilated cardiomyopathy. *Biochemical and Biophysical Research Communications*.

[103] Murakami T., Hayashi Y. K., Noguchi S. (2006). Fukutin gene mutations cause dilated cardiomyopathy with minimal muscle weakness. *Annals of Neurology*.

[104] Müller T., Krasnianski M., Witthaut R., Deschauer M., Zierz S. (2005). Dilated cardiomyopathy may be an early sign of the C826A Fukutin-related protein mutation. *Neuromuscular Disorders*.

[105] Minoretti P., Arra M., Emanuele E. (2007). A W148R mutation in the human FOXD4 gene segregating with dilated cardiomyopathy, obsessive-compulsive disorder, and suicidality. *International Journal of Molecular Medicine*.

[106] Theis J. L., Sharpe K. M., Matsumoto M. E. (2011). Homozygosity mapping and exome sequencing reveal GATAD1 mutation in autosomal recessive dilated cardiomyopathy. *Circulation: Cardiovascular Genetics*.

[107] Meder B., Rühle F., Weis T. (2014). A genome-wide association study identifies 6p21 as novel risk locus for dilated cardiomyopathy. *European Heart Journal*.

[108] Pankuweit S., Ruppert V., Jónsdóttir T., Müller H.-H., Meyer T. (2013). The HLA class II allele DQB1^*^0309 is associated with dilated cardiomyopathy. *Gene*.

[109] Stark K., Esslinger U. B., Reinhard W. (2010). Genetic association study identifies *HSPB7* as a risk gene for idiopathic dilated cardiomyopathy. *PLoS genetics*.

[110] Knöll R., Postel R., Wang J. (2007). Laminin-alpha4 and integrin-linked kinase mutations cause human cardiomyopathy via simultaneous defects in cardiomyocytes and endothelial cells. *Circulation*.

[111] Carboni N., Marrosu G., Porcu M. (2011). Dilated cardiomyopathy with conduction defects in a patient with partial merosin deficiency due to mutations in the laminin-*α*2-chain gene: a chance association or a novel phenotype?. *Muscle & Nerve*.

[112] Maron B. J., Roberts W. C., Arad M. (2009). Clinical outcome and phenotypic expression in LAMP2 cardiomyopathy. *The Journal of the American Medical Association*.

[113] Arimura T., Hayashi T., Terada H. (2004). A Cypher/ZASP mutation associated with dilated cardiomyopathy alters the binding affinity to protein kinase C. *The Journal of Biological Chemistry*.

[114] Fatkin D., Macrae C., Sasaki T. (1999). Missense mutations in the rod domain of the lamin A/C gene as causes of dilated cardiomyopathy and conduction-system disease. *The New England Journal of Medicine*.

[115] Rodriguez G., Ueyama T., Ogata T. (2011). Molecular genetic and functional characterization implicate muscle-restricted coiled-coil gene (MURC) as a causal gene for familial dilated cardiomyopathy. *Circulation: Cardiovascular Genetics*.

[116] Daehmlow S., Erdmann J., Knueppel T. (2002). Novel mutations in sarcomeric protein genes in dilated cardiomyopathy. *Biochemical and Biophysical Research Communications*.

[117] Hershberger R. E., Norton N., Morales A., Li D., Siegfried J. D., Gonzalez-Quintana J. (2010). Coding sequence rare variants identified in MYBPC3, MYH6, TPM1, TNNC1, and TNNI3 from 312 patients with familial or idiopathic dilated cardiomyopathy. *Circulation: Cardiovascular Genetics*.

[118] Carniel E., Taylor M. R. G., Sinagra G. (2005). *α*-myosin heavy chain: a sarcomeric gene associated with dilated and hypertrophic phenotypes of cardiomyopathy. *Circulation*.

[119] Duboscq-Bidot L., Xu P., Charron P. (2008). Mutations in the Z-band protein myopalladin gene and idiopathic dilated cardiomyopathy. *Cardiovascular Research*.

[120] Purevjav E., Varela J., Morgado M. (2010). Nebulette mutations are associated with dilated cardiomyopathy and endocardial fibroelastosis. *Journal of the American College of Cardiology*.

[121] Hassel D., Dahme T., Erdmann J. (2009). Nexilin mutations destabilize cardiac Z-disks and lead to dilated cardiomyopathy. *Nature Medicine*.

[122] Matsa L. S., Rangaraju A., Vengaldas V. (2013). Haplotypes of NOS3 gene polymorphisms in dilated cardiomyopathy. *PLoS ONE*.

[123] Haghighi K., Kolokathis F., Pater L. (2003). Human phospholamban null results in lethal dilated cardiomyopathy revealing a critical difference between mouse and human. *Journal of Clinical Investigation*.

[124] Arndt A.-K., Schafer S., Drenckhahn J.-D. (2013). Fine mapping of the 1p36 deletion syndrome identifies mutation of PRDM16 as a cause of cardiomyopathy. *American Journal of Human Genetics*.

[125] Li D., Parks S. B., Kushner J. D. (2006). Mutations of presenilin genes in dilated cardiomyopathy and heart failure. *The American Journal of Human Genetics*.

[126] Brauch K. M., Karst M. L., Herron K. J. (2009). Mutations in ribonucleic acid binding protein gene cause familial dilated cardiomyopathy. *Journal of the American College of Cardiology*.

[127] Bhuiyan Z. A., van den Berg M. P., van Tintelen J. P. (2007). Expanding spectrum of human RYR2-related disease: new electrocardiographic, structural, and genetic features. *Circulation*.

[128] McNair W. P., Ku L., Taylor M. R. G. (2004). SCN5A mutation associated with dilated cardiomyopathy, conduction disorder, and arrhythmia. *Circulation*.

[129] Levitas A., Muhammad E., Harel G. (2010). Familial neonatal isolated cardiomyopathy caused by a mutation in the flavoprotein subunit of succinate dehydrogenase. *European Journal of Human Genetics*.

[130] Tsubata S., Bowles K. R., Vatta M. (2000). Mutations in the human *δ*-sarcoglycan gene in familial and sporadic dilated cardiomyopathy. *Journal of Clinical Investigation*.

[131] Zhang Q., Bethmann C., Worth N. F. (2007). Nesprin-1 and -2 are involved in the pathogenesis of Emery–Dreifuss muscular dystrophy and are critical for nuclear envelope integrity. *Human Molecular Genetics*.

[132] Bione S., D'Adamo P., Maestrini E., Gedeon A. K., Bolhuis P. A., Toniolo D. (1996). A novel X-linked gene, G4.5. is responsible for Barth syndrome. *Nature Genetics*.

[133] Kirk E. P., Sunde M., Costa M. W. (2007). Mutations in cardiac T-box factor gene TBX20 are associated with diverse cardiac pathologies, including defects of septation and valvulogenesis and cardiomyopathy. *American Journal of Human Genetics*.

[134] Hayashi T., Arimura T., Itoh-Satoh M. (2004). Tcap gene mutations in hypertrophic cardiomyopathy and dilated cardiomyopathy. *Journal of the American College of Cardiology*.

[135] Taylor M. R. G., Slavov D., Gajewski A. (2005). Thymopoietin (lamina-associated polypeptide 2) gene mutation associated with dilated cardiomyopathy. *Human Mutation*.

[136] Murphy R. T., Mogensen J., Shaw A., Kubo T., Hughes S., McKenna W. J. (2004). Novel mutation in cardiac troponin I in recessive idiopathic dilated cardiomyopathy. *The Lancet*.

[137] Kamisago M., Sharma S. D., DePalma S. R. (2000). Mutations in sarcomere protein genes as a cause of dilated cardiomyopathy. *The New England Journal of Medicine*.

[138] Sibbing D., Pfeufer A., Perisic T. (2011). Mutations in the mitochondrial thioredoxin reductase gene TXNRD2 cause dilated cardiomyopathy. *European Heart Journal*.

[139] Gerull B., Gramlich M., Atherton J. (2002). Mutations of TTN, encoding the giant muscle filament titin, cause familial dilated cardiomyopathy. *Nature Genetics*.

[140] Olson T. M., Illenberger S., Kishimoto N. Y., Huttelmaier S., Keating M. T., Jockusch B. M. (2002). Metavinculin mutations alter actin interaction in dilated cardiomyopathy. *Circulation*.

[141] Li X., Luo R., Mo X. (2013). Polymorphism of ZBTB17 gene is associated with idiopathic dilated cardiomyopathy: a case control study in a Han Chinese population. *European Journal of Medical Research*.

[142] Andreasen C., Nielsen J. B., Refsgaard L. (2013). New population-based exome data are questioning the pathogenicity of previously cardiomyopathy-associated genetic variants. *European Journal of Human Genetics*.

[143] Philipp U., Vollmar A., Distl O. (2008). Evaluation of six candidate genes for dilated cardiomyopathy in Irish wolfhounds. *Animal Genetics*.

[144] Lynne O'Sullivan M., O'Grady M. R., Glen Pyle W., Dawson J. F. (2011). Evaluation of 10 genes encoding cardiac proteins in Doberman Pinschers with dilated cardiomyopathy. *American Journal of Veterinary Research*.

[145] Stabej P., Leegwater P. A. J., Imholz S. (2005). The canine sarcoglycan delta gene: BAC clone contig assembly, chromosome assignment and interrogation as a candidate gene for dilated cardiomyopathy in Dobermann dogs. *Cytogenetic and Genome Research*.

[146] Philipp U., Vollmar A., Distl O. (2008). Evaluation of the *Titin-Cap Gene* (*TCAP*) as candidate for dilated cardiomyopathy in Irish wolfhounds. *Animal Biotechnology*.

[147] Oyama M. A., Chittur S. (2005). Genomic expression patterns of cardiac tissues from dogs with dilated cardiomyopathy. *American Journal of Veterinary Research*.

